# Preeclampsia Prediction and Management

**DOI:** 10.1155/2014/502081

**Published:** 2014-11-09

**Authors:** Irene Rebelo, João Bernardes

**Affiliations:** ^1^Department of Biochemistry, Faculty of Pharmacy, University of Porto, Rua de Jorge Viterbo Ferreira 228, 4050-313 Porto, Portugal; ^2^Institute for Molecular and Cell Biology (IBMC), University of Porto, 4150-180 Porto, Portugal; ^3^Faculty of Medicine, University of Porto, 4200-319 Porto, Portugal; ^4^Center for Research in Health Technologies and Information Systems (CINTESIS), Faculty of Medicine, University of Porto, 4200-450 Porto, Portugal; ^5^Department of Obstetrics and Gynecology, Pedro Hispano Hospital, 4454-509 Matosinhos, Portugal; ^6^Department of Obstetrics and Gynecology, S. João Hospital, 4200-450 Porto, Portugal

International guidelines still simply define preeclampsia (PE) as an acute pregnancy related hypertensive condition characterized by hypertension and proteinuria that typically appears after the 20 weeks of gestation and resumes after delivery [1]. With these relatively simple guidelines centered on blood pressure and proteinuria assessment, along with eclampsia prevention with magnesium sulphate and fetal delivery in the most severe cases, the developed countries have managed to control the high maternal and fetal mortality rates related with PE that still affect the developing countries without adequate basic clinical ante- and intrapartum facilities [1].

However, we know today that PE is a more complex condition that develops during the first weeks of pregnancy and that may have consequences in the future health of the mother and child.

PE remains a leading cause not only of maternal and fetal mortality in the developing countries, but also of morbidity in the developed countries accounting for a high number of maternal admissions to intensive care units, fetal growth restriction, and premature iatrogenic deliveries, without effective early prediction and/or prevention. Moreover, with the increased life expectancy of the developed countries it is also known today that women with history of PE and their offspring present an increased risk of future hypertension and cardiovascular diseases, among others [1].

In this special issue, several authors address the above-mentioned issues, namely, on early PE prediction, management, and risk of future cardiovascular diseases [2].

L. C. Poon and K. H. Nicolaides remind us that PE screening by a combination of maternal risk factors, uterine artery Doppler, mean arterial pressure, maternal serum pregnancy associated plasma protein-A, and placental growth factor can identify about 95% of cases of early onset PE for a false-positive rate of 10%. This excellent news can be already put in practice using specially commercialized kits. It opens new perspectives on early prediction and diagnosis, allowing better application of preventive and curative measures, namely, using, respectively, aspirin and timely antihypertensive treatment and/or pregnancy termination [1]. This hope for better perspectives on early prediction of PE has also been exposed by C. Teixeira et al., who managed to show that even a common program for first trimester screening of aneuploidies may already improve our current capabilities based only on the relatively soft above-mentioned clinical assessment of blood pressure and proteinuria [1], although in a much more modest way than when using the model presented by L. C. Poon and K. H. Nicolaides.

On the other hand, S. C. Kane et al. elaborate on contemporary management principles pertaining to maternal and fetal neurological sequelae of PE. As they outline, the neurological complications of preeclampsia and eclampsia are major contributors of PE related maternal and fetal morbidity and mortality that need to be seriously taken into account and adequately addressed.

Finally, A. Matos et al. and P. V. Pinto et al. tackle the issue of PE and the risk of future cardiovascular disease. A. Matos et al. concluded that previously PE women, either subsequently hypertensive or normotensive, present significant differences in myeloperoxidase, nitrites, liver enzymes, and other cardiovascular risk biomarkers, whose variation may be modulated by haptoglobin 1/2 functional genetic polymorphism. They provide more evidence not only on the association between PE and future cardiovascular diseases, but also on the putative pathogenic paths underlying this situation. However, in contrast with all these developments on the recognition and understanding of the association between PE and the development of future cardiovascular disease, P. V. Pinto et al. showed that the majority of 141 cases of preeclampsia and chronic hypertension with superimposed preeclampsia diagnosed at their institution between January 2010 and December 2013, as well as general practitioners, did not take into consideration a previous pregnancy affected by preeclampsia as a risk factor for future cardiovascular disease, namely, in the implementation of healthy behaviours and/or adequate medical treatment. This shows that educational and prevention programs urge in this area, in both patients and the general practitioners levels.

We hope this special issue provides not only new data for daily clinical practice, but also inspiration to pursue the hard way of PE research, in all its multiple and complex areas.

*Irene Rebelo* *Irene Rebelo* *João Bernardes* *João Bernardes*
